# Effectiveness of Anti-tumor Necrosis Factor Drugs on Hidradenitis Suppurativa: A Systematic Review

**DOI:** 10.7759/cureus.74172

**Published:** 2024-11-21

**Authors:** Rojina Samifanni, Vincent Gatt, Jessica Kabore, Mariangela Silva, Manal Khan, Theofanis F Kollias, Lucy A Clunes

**Affiliations:** 1 Dermatology, St. George's University School of Medicine, St. George's, GRD; 2 Microbiology, Immunology, and Pharmacology, St. George's University School of Medicine, St. George's, GRD; 3 Pharmacology, St. George's University School of Medicine, St. George's, GRD

**Keywords:** adalimumab, anti-il-17, anti-tnf, etanercept, hidradenitis suppurativa, il-17

## Abstract

Hidradenitis suppurativa (HS) is a painful and chronic inflammatory skin disease with no consistently effective treatment, affecting a significant portion of the Western population. HS is characterized by painful nodules, abscesses, tunnels, and scarring in body folds. The immunobiology is poorly understood, therefore resulting in a lack of effective therapies. Despite indications of microbial involvement, antimicrobial treatments have shown inconsistent and temporary results. Recent studies have identified dysregulated immune responses as a key factor in HS. This has led to the use of biologic agents, most notably adalimumab, which is currently the only FDA-approved therapy for HS. Due to the limited understanding of immune dysregulation in HS, ongoing clinical trials are adapting treatments from other skin conditions, such as psoriasis. As a result, researchers look to other skin conditions with better-understood pathophysiologies, such as psoriasis, as a starting point for developing treatments for HS. While adaptation can offer some benefits, such as immediate treatment options, the lack of specificity can lead to side effects that are not well tolerated, and long-term efficacy may be uncertain. Therefore, there is a pressing need for a foundational understanding of HS's immune dysregulation. Current ongoing research is exploring other options, such as therapies targeting IL-17, in addition to anti-TNF therapies, which have shown the potential to be more effective. With new options emerging, it is essential to evaluate the current performance of anti-TNF drugs in treating HS. This review was conducted to thoroughly assess the effectiveness of these therapies, offering a detailed analysis to inform future research and clinical practice. The objective is to determine whether anti-TNF drugs continue to be a strong treatment option or if newer therapies might lead to better outcomes for patients.

## Introduction and background

Hidradenitis suppurativa (HS)

HS is an inflammatory skin disease characterized by the formation of multiple abscesses and nodules in the apocrine gland-bearing areas. It is localized primarily in the intertriginous areas such as the groin, axilla, gluteal cleft, and inframammary folds [1]. Acne inversa is another name for the condition, which advances through various stages, from inflammatory nodules to severe tunneling and scarring. Typically, HS first appears in early adulthood, peaking in the third decade of life. Studies show that both men and women can be affected, though a higher proportion of affected women have been reported. A positive family history is associated with a higher prevalence of the illness, suggesting a possible genetic predisposition [2].

Pathogenesis of HS

The etiology and pathogenesis of HS are unknown, but various factors are believed to play a role. The pathogenesis of HS involves a complex interplay of genetic, environmental, and immunological factors. While the exact cause remains elusive, it is widely accepted that follicular occlusion, inflammation, and abnormal immune responses contribute to the development of the condition. The disease often starts with hair follicle blockage, leading to the formation of comedones, which subsequently progress to inflammatory nodules and abscesses. Dysregulation of the immune system, including abnormalities in innate and adaptive immune responses, further exacerbates the inflammatory process. Genetic factors, such as a family history of HS, also play a role, suggesting a genetic predisposition to the condition.

Although evidence is variable, some studies show that hormones, obesity, and smoking can also aggravate HS. Patients diagnosed with HS experience a reduction in quality of life due to the lesions and scarring. Quality of life scores with HS are lower than those who have been reported for psoriasis. HS is often difficult to treat, and most therapeutic options usually target factors related to genetic and environmental influences. Medical treatments, including various antibiotics and oral contraceptives, have been recommended for HS but have yielded limited efficacy. Numerous trials with certain medications, such as topical clindamycin and systemic tetracycline or anti-androgen therapies, have failed, leading to surgery becoming the therapy of choice. More contemporary research led to the discovery of biological agents as pro-inflammatory mediators. In recent years, biological agents such as tumor necrosis factor (TNF)-α inhibitors have been introduced, with evidence showing efficacy for the treatment of HS [3].

Role of anti-TNF drugs on HS

TNF-α is a pro-inflammatory cytokine produced by macrophages and monocytes but can also be found in the basal layer of the epidermis, sweat glands, and hair follicles. Upon binding to specific receptors, TNF-α triggers an intracellular cascade that leads to a pro-inflammatory response. Targeting TNF-α disrupts this cascade at a critical point in HS. It was reported that TNF inhibitors proved to be 85% effective, with 6% of patients not responding to the medications and 7% experiencing side effects when treated with infliximab, adalimumab, and etanercept. The side effects were similar to those reported in other TNF-α studies for different diseases. One important factor noted was that 14% of patients were in remission [1]. This suggests that TNF inhibitors are generally effective in managing HS. However, there is variability in patient response. This highlights the need for personalized treatment approaches and further research to optimize therapeutic strategies for those who do not respond adequately or experience adverse effects.

Aim

It is well known that most cases of HS are characterized by painful abscesses and nodules, recurrent boils, and scarring. The management of HS involves a diverse array of treatments aimed at alleviating symptoms and improving the quality of life for affected individuals. Common approaches include topical treatments, such as antibiotics and corticosteroids, or systemic medications. This paper aims to identify gaps in the current literature and provide insights into the variability in patient responses to anti-TNF therapy, which may inform future research directions and clinical practice.

## Review

Methods

The review was carried out using literature from 2013 to 2023 through two search engines: Google Scholar and PubMed. The keywords used during the search were: “anti-TNF drugs on HS”, “TNF-α inhibitors”, “anti-TNF treatment hidradenitis suppurativa”, “retinoid treatment hidradenitis suppurativa”, “hidradenitis suppurativa”, “TNF-α”, and “anti-TNF therapy”. The research focused on publications that discussed experimental results as well as observations noted during the process. Publications were included in the review based on their availability and relevance to anti-TNF treatment in HS - those that deviated from the topic of anti-TNF treatment focused on alternative medications and their effects on HS. Before narrowing down the publications, both databases yielded a total of 3,540 articles, from which 13 publications were ultimately selected.

Inclusion Criteria

The publications used in this paper were chosen based on the following inclusion criteria: publications between 2010 and 2023, performed on humans or cell cultures, focused on the effectiveness of anti-TNF drugs as a treatment regime for HS, as well as other remedies that have been shown to be more effective, and peer-reviewed experimental studies. Filters were used to include clinical and randomized trials. 

Exclusion Criteria

**Figure 1:**
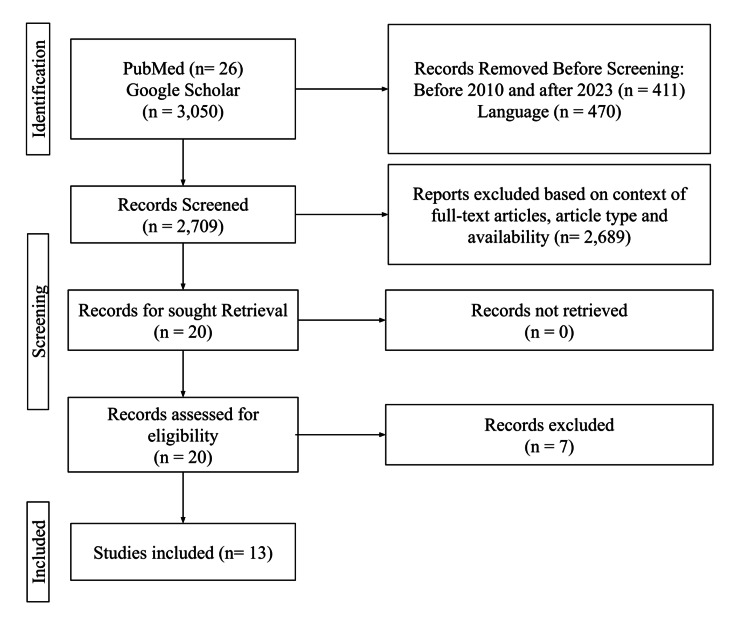
Identification of the inclusion and exclusion criteria progression

The publications were excluded based on the following criteria: review articles, any articles prior to 2010, and any papers in languages other than English and were filtered based on relativity to the main area of interest. Papers that included other diseases and did not focus on HS were also excluded. The specifics of the selection of publications are based on set inclusion and exclusion criteria, which are indicated in Figure 1. 

Bias

All publications were assessed for bias via the Grading of Recommendations Assessment, Development, and Evaluation (GRADE) scale, resulting in a moderate bias rating.

Results

A total of 3,540 publications were discovered: 3,470 were from Google Scholar, and 70 were from PubMed. Among the exclusions, 411 were removed as they were published before 2010 or after 2023, and 470 were in another language other than English. A total of 831 publications were excluded during the automatic screening procedure, leading to 2,709 publications for manual screening. Publications were then manually evaluated based on their full-text accessibility, title, and type of study. Thirteen publications were selected. The results were based on an analysis of selected articles, summarized below in Table 1.

**Table 1:** Summary of articles analyzed in this review APRIL, A proliferation-inducing ligand; BAFF, B cell-activating factor; CSF1, colony-stimulating factor 1; GM-CSF, granulocyte-macrophage colony-stimulating factor; HS, hidradenitis suppurativa; IFN, interferon; IFN-γ, interferon gamma; IFNLR1, type I interferon receptor; mTOR, mammalian target of rapamycin; mTORC1, mammalian target of rapamycin complex 1; TNF-α, tumor necrosis factor-alpha

Author(s)	Journal	Study Population	Finding(s)	Conclusion(s)
Lowe et al., 2020 [4]	JCI Insight	Case-control study (n = 48)	Lesions of HS patients had dysfunctional dendritic cells, a reduced quantity of regulatory T cells, and a more significant influx of memory B cells, plasma cells, and neutrophils. Lesional skin showed increased levels of TNF-α-regulated genes, IFN-γ, IL-1β, IL-1RN (IL-1 receptor antagonist), IL-10RA, CXCL13, CXCR5, BAFF (B cell survival factor), and APRIL (proliferation-inducing ligand). In non-lesional HS skin, there was a decrease in dendritic cells, activated M2 macrophages, CD163 (immunoregulatory macrophage marker), and IL-1R2 (negative regulator of IL-1 signaler). However, there was an increase in TNF-α, CSF1, and IFNLR1 (type I IFN receptor) levels. After treatment with anti-TNF-α, B cell pathway genes, complement activation, phagocytosis, memory B cell quantity, plasma cell quantity, CXCL13 levels, and BAFF levels were markedly decreased.	Healthy non-lesional skin in HS patients is functionally different from healthy non-lesional skin in healthy subjects regarding their immune responses and regulatory pathways. Anti-TNF-α therapy reduced B cell activation without significantly altering other inflammatory pathways.
Moran et al., 2017 [5]	Journal of Investigative Dermatology	Case-control study (n = 26)	HS lesions had a greater amount of regulatory T cells and inflammatory cytokines from T helper type 17 (Th17) T cells producing IL-17 cytokines. These Th17 cells also produced a variety of other proinflammatory cytokines (GM-CSF, IL-22, IFN-γ) in addition to TNF. Anti-TNF therapy significantly reduced the amount of Th17 cells in lesions, bringing the ratio of Th17 T cells to regulatory T cells closer to what is expected in a healthy patient’s skin.	Suggests that inhibition of IL-17 production via anti-TNF therapy will improve immunological dysregulation found in lesions of HS patients.
Balato et al., 2018 [6]	Journal of the European Academy of Dermatology and Venereology	Prospective cohort study (n = 20)	There is an observed increase in mTOR expression in the affected and unaffected skin of HS patients, which directly correlates with disease severity. mTOR activation stimulates the secretion of steroids and promotes the adhesion of hair follicles, leading to follicular plugging. Adalimumab treatment has been shown to significantly reduce mTOR1 expression. Study results indicate that anakinra therapy has proven to be safe as an alternative HS treatment for patients, with injection site pain and mild infections being within the drug’s safety profile.	Anti-TNF-α therapy is effective in treating HS, in part due to its ability to modulate the mTORC1 signaling pathway. This downregulation of mTORC1 may help to reduce inflammation and cellular proliferation associated with HS.
Fotiadou et al., 2016 [7]	Clinical Cosmetology and Investigative Dermatology	Case-control study (n = 42)	Clinical responses were more significant in the treatment group receiving adalimumab (monoclonal antibody used as anti-TNF-α therapy) compared to the placebo group.	Suggests that the proposed adverse effects of adalimumab are more likely to be symptoms of HS.
Kimball et al., 2016 [8]	The New England Journal of Medicine	307 patients in PIONEER I and 326 in PIONEER II	Longitudinal and case studies focused on the therapeutic potential of adalimumab (monoclonal anti-TNF-α antibody) have determined that more than half of severe HS patients will have symptom alleviation after treatment (reduction in the number of affected areas); however, the relapse rate is just as high if patients decide to discontinue treatment.	Treatment with adalimumab (40 mg weekly), as compared with placebo, resulted in significantly higher clinical response rates in both trials at 12 weeks. It was efficacious for the treatment of moderate-to-severe HS, and the majority of adverse events were mild or moderate, with no evidence of an increased risk of serious adverse events with adalimumab as compared with placebo.
Prens et al., 2021 [9]	British Journal of Dermatology	Retrospective cohort study (n = 104)	After 12 weeks of treatment, adalimumab has a HiSCR rate of 41.8% and 58.9%. Long-term follow-up demonstrated that this response rate was maintained at 52.3%. Older age, higher BMI, severity of HS, and longer disease duration increased the survival rate of adalimumab.	This study suggests that a combination of both biological and surgical therapies can optimize treatments and results for patients with HS.
Wohlmuth-Wieser et al., 2021 [10]	International Journal of Dermatology	Case report	Certolizumab pegol was chosen because it does not have the Fc region that typically facilitates placental transfer, making it potentially safer during pregnancy. The patient showed significant improvement in HS symptoms during pregnancy while being treated with certolizumab pegol. Her pain levels decreased, and there was a reduction in the number of abscesses and inflammatory nodules.	The study concluded that certolizumab pegol can be an effective and safe treatment for severe HS in pregnant patients, providing significant symptom relief. The medication was well-tolerated with no adverse effects on the pregnancy or the infant, highlighting its potential safety profile.
Adams et al., 2010 [11]	Archives of Dermatology	Cross-over clinical trial (n = 20)	Etanercept (a dimeric human TNF receptor) was discussed regarding its potential use as an HS therapy. However, no significant clinical responses were found between cohorts after 12 and 24 weeks, despite earlier studies that conveyed its clinical effectiveness.	Although well tolerated by patients, subcutaneous administration of etanercept did not demonstrate a significant improvement in HS.
Martin-Ezquerra et al., 2017 [12]	Italian Dermatology and Venereology	Meta-analysis	HS patients are likely to experience “flare-ups” of symptoms regardless of their treatment modality and discontinuing any of these medications would result in a relatively quick relapse of disease symptoms. Only ~60% of HS patients using adalimumab (anti-TNF therapy) have clinical responses without adverse effects, and only 50% of infliximab (anti-TNF therapy) users experience reductions in lesion size and/or severity. However, ustekinumab (anti-TNF therapy) achieved symptom improvement rates as high as 82%.	Suggests that HS has multifactorial pathology, which likely requires more than anti-TNF therapy, such as antibiotics or retinoids, to achieve complete symptom reduction and prevent relapse from occurring.
Schell et al., 2023 [13]	British Journal of Dermatology	87 patients with HS and 39 healthy controls	Keratinocytes were identified as the main source of inflammatory cytokines in HS lesions, with limited contribution from dermal immune cells. When exposed to IFN-γ, keratinocytes expressed even more cytokines, including IL-1β, IL-12, IL-23, and IL-36γ.	Keratinocytes actively recruit immune cells to HS epidermis. The JAK inhibitor ruxolitinib reduced the expression of inflammatory cytokines and chemokines in HS lesional keratinocytes, suggesting its potential as a topical treatment for HS, which could offer more effective results with fewer side effects.
Boer et al., 2011 [14]	British Journal of Dermatology	Retrospective cohort study (n = 12)	Long-lasting improvement was conveyed by nine of the 12 affected patients as they did not have symptom recurrence after six months (n = 1), one year (n = 3), >2 years (n = 2), >3 years (n = 2), and >4 years (n = 1), while the remaining three patients demonstrated moderate improvement. Deep nodules and abscesses were absent post-treatment, and there was a significant decrease in comedones, leaving only non-inflamed superficial nodules or pimples.	Post-treatment, nine of the 12 patients had long-lasting improvement and or remission following discontinuation of acitretin (remissions ranged from six to 45 months), suggesting that acitretin not only causes symptom relief and remission but that, in most cases, the effects can be long-lasting post-treatment, unlike most other available HS therapies.
Glatt et al., 2021 [15]	JAMA Dermatology	Double-blind, placebo-controlled randomized clinical trial	Bimekizumab (another anti-IL-17 agent) is also undergoing phase III clinical trials and is about as effective as secukinamub in terms of its clinical response rates going beyond 70% after 12 weeks. However, bimekizumab can reduce two types of interleukins (IL-17A and IL-17F), while secukinumab will only reduce IL-17A levels. This dual inhibitory effect makes bimekizunab seem more promising for HS treatment, and it consistently outperforms any placebo group outcome.	The positive results suggest that bimekizumab could become a valuable treatment option for this condition, addressing an unmet medical need. Further research in larger clinical trials is recommended to confirm these findings and evaluate long-term safety and efficacy.
Tzanetakou et al., 2016 [16]	JAMA Dermatology	Double-blind, randomized, placebo-controlled clinical trial	12-week treatment phase and follow-up phase for patients with HS and Hurley stage II or III HS. Patients were monitored at baselines, with six evaluations performed at each visit. The disease activity score decreased in 20% of placebo-treated patients compared to 78% of anakinra-treated patients at week 12. The HiSCR components were positive in 30% of patients randomized to placebo compared to 78% of those randomized to anakinra.	Anakinra therapy has proven to be safe as an alternative HS treatment for patients, with injection site pain and mild infections being within the drug’s safety profile

Discussion

Lowe et al. (2020) demonstrated that healthy non-lesional skin in HS patients differs functionally from that of healthy subjects. Specifically, the immune responses and regulatory pathways in HS patients' skin are somewhat dysfunctional. Additionally, it was determined that lesions of HS patients had dysfunctional dendritic cells, a reduced quantity of regulatory T cells, and a more significant influx of memory B cells compared to healthy controls. Furthermore, plasma cell infiltration in these lesions appeared to be more prevalent, such that the amount of cells infiltrating is higher than expected. Myeloid cells in HS lesions have higher rates of IL-1-related inflammatory responses. 

Analysis of HS lesional skin showed increased levels of TNF-α-regulated genes, interferon gamma (IFN-γ), and IL-1β with reduced levels of IL-1RN (IL-1 receptor antagonist) and IL-10RA. In non-lesional HS skin, there was a decrease in dendritic cells and activated M2 macrophages, a cluster of differentiation 163 (CD163) (immunoregulatory macrophage marker), and IL-1R2 (a negative regulator of IL-1 signaler). However, there was an increase in TNF-α, colony-stimulating factor 1 (CSF1), and type I interferon (IFN) receptor (IFNLR1) levels. HS skin showed increased levels of neutrophils. In HS skin lesions, increased levels of CXCL13, CXCR5, B cell survival factor (B cell-activating factor (BAFF)), and A proliferation-inducing ligand (APRIL) were seen. After treatment with anti-TNF-α, B cell pathway genes, complement activation, and phagocytosis were all decreased. Significant decreases were seen in memory B cells and plasma cells. CXCL13 and BAFF levels were markedly decreased. Diminished B-cell-specific gene expression were seen. Neutrophil activation, type I IFN production, IFN-γ production, and IL-1β secretion remained unchanged after treatment. Leukocyte chemotaxis, T cell cytokine production regulation of B cell proliferation, and IL-6/IL-8 expression were seen to be elevated in patients who were unresponsive to anti-TNF-α treatment. CXCL6, CXCR1, CCL17, CCR7, CXCR4, CD19, CSCR5, BAFF, and IL-1α were shown to be elevated in unresponsive patients with increased and greater increases in IL-1β. 

In contrast, in patients who responded to treatment, there was seen to be an increase in epithelial skin development and keratinocyte differentiation pathways with decreased inflammatory signatures. Anti-TNF-α therapy reduced B cell activation without significantly altering other inflammatory pathways [4].

Moran et al. (2017) focused on the effects of HS pathology on skin-infiltrating T-cell responses. It was determined that HS lesions had a disproportionate amount of inflammatory cytokines from T helper type 17 (Th17) T cells producing IL-17 cytokines. These cells also produced a variety of other pro-inflammatory cytokines (granulocyte-macrophage colony-stimulating factor (GM-CSF), IL-22, IFN-γ) in addition to TNF. Additionally, a greater quantity of regulatory T cells was found in HS lesions compared to controls. However, this difference is minuscule compared to the disproportionate amount of Th17 T cells. Anti-TNF therapy significantly reduced the amount of Th17 T cells in lesions, bringing the ratio of Th17 T cells to regulatory T cells closer to what is expected in a healthy patient’s skin. This finding suggests that inhibition of IL-17 production via anti-TNF therapy will improve immunological dysregulation found in lesions of HS patients [5].

HS is characterized by a significant increase in pro-inflammatory substances like IFN-γ, TNF-α, IL-1, IL-17, and IL-12/23 [5]. Balato et al. (2019) state that the most promising biologics in ongoing phase III trials include anti-IL-17 antibodies like secukinumab and bimekizumab. Additionally, a biologic targeting anti-IL-1, known as bermekimab, is showing promising results in phase II trials. Balato et al. (2019) also describe HS as being correlated with γ-secretase, an intramembrane protease complex comprising four hydrophobic proteins: presenilin (PSEN), presenilin enhancer-2 (PSENEN), nicastrin (NCSTN), and anterior pharynx defective 1 (APH1). γ-secretase is responsible for canonical Notch signaling, a process crucial for keratinocyte maturation and differentiation. Mutations in NCSTN, PSEN1, or PSENEN genes lead to impaired γ-secretase activity, resulting in deficient Notch signaling. This leads to the accumulation of keratin in hair follicles, forming plugs. Furthermore, Balato et al. (2019) mention an observed increase in mammalian target of rapamycin (mTOR) expression in the affected and unaffected skin of HS patients, which directly correlates with disease severity. mTOR activation stimulates the secretion of steroids and promotes the adhesion of hair follicles, leading to follicular plugging. Adalimumab treatment has been shown to significantly reduce mTOR1 expression [6].

Adalimumab

Currently, adalimumab, a monoclonal anti-TNF-α antibody, is the only approved treatment for patients with moderate-severe HS symptoms. In a study performed by Fotiadou et al. (2016), 42 severe case reports were considered to determine that ~60% of severe HS patients exhibit clinical responses, but ~70% relapse once treatment is discontinued. However, the study also compared the drug's effectiveness with that of a placebo group. The results showed that the supposed adverse effects of adalimumab were similar to those experienced by the placebo group. This suggests that the proposed adverse effects are more likely to be symptoms of HS. Furthermore, as expected, clinical responses (reduction in the number of affected areas, nodules, or fistulas) were much more significant in the treatment group. It should be noted that adalimumab treatment of HS has the most extensive data pool compared to any other treatment modality. However, larger-scale studies must be undertaken to establish this treatment's true efficacy and safety [7].

Kimball et al. (2016) showed that clinical response rates at week 12 were significantly higher for the groups receiving adalimumab weekly than for the placebo groups: 41.8% versus 26.0% in PIONEER I (p = 0.003) and 58.9% versus 27.6% in PIONEER II (p < 0.001). Treatment with adalimumab (40 mg weekly), as compared with placebo, resulted in significantly higher clinical response rates in both trials at 12 weeks; rates of serious adverse events were similar in the study groups. In both phase 3 studies, the results at week 12 confirmed the findings of the phase 2 trial. They showed that 40 mg of adalimumab weekly was effective for treating moderate-to-severe HS. The majority of adverse events were mild or moderate, with no evidence of an increased risk of serious adverse events when compared to placebo. These two randomized trials involving patients with moderate-to-severe HS demonstrated that adalimumab significantly increased the likelihood of a clinically meaningful response at week 12. This response was defined as at least a 50% reduction from baseline in the total abscess and inflammatory-nodule count, with no increase in abscess or draining-fistula counts. This effect was observed with or without continued antibiotic treatment [8].

The goal of a study by Prens et al. (2021) aimed to assess drug survival and identify factors associated with drug survival in daily practice settings. Adalimumab was found to have Hidradenitis Suppurativa Clinical Response (HiSCR) achievement rates of 41.8% and 58.9% at week 12. Long-term follow-up demonstrated that this response was maintained in 52.3% of patients. The study included patients diagnosed with HS who had received previous treatments with anti-TNF-⍺. An analysis of these patients was done to assess the outcomes and influences of the previous treatments, but the data were not significant for previous treatments. Data on age, sex, BMI disease severity, smoking status, treatment history, comorbidities, and family history of HS were collected. The study also analyzed data on the type of biological agent, use of biosimilars, treatment regimens, start and stop dates, reasons for discontinuation, concomitant medication, and side effects. Adalimumab’s median overall drug survival is 18.1 months. The overall survival rate for adalimumab at 12 months was 56% and 31% at 24 months. The main reasons for discontinuing the treatment were ineffectiveness, side effects, and remission following surgical interventions. Drug survival rates for adalimumab were observed to be lower than in other HS-related immune diseases. Older age and longer disease duration were found to be significant predictors of increased survival for adalimumab, as well as moderate or severe HS. Patients with higher BMI also demonstrated significantly longer survival of adalimumab. Results of the study suggest that a combination of both biological and surgical therapies can optimize treatments and results for patients with HS [9].

Wohlmuth-Wieser et al. (2021) reported a case that described the successful treatment of a 34-year-old patient with HS using certolizumab pegol (CZP), an anti-TNF-α medication that lacks the fragment crystallizable (Fc) region preventing active placental transfer. The patient initially had severe HS, which was managed with antibiotics and adalimumab, resulting in significant improvement. However, during her pregnancy, she decided to discontinue adalimumab due to concerns about its effects on her baby. Subsequently, she experienced an HS flare at 19 weeks of gestation and began off-label CZP treatment. CZP was well-tolerated and effectively controlled her symptoms. Her condition improved significantly, and she delivered a healthy baby at 40 weeks of gestation. Post-pregnancy, she decided to switch back to adalimumab [10].

Up until 2023, adalimumab is currently the only FDA-approved treatment for moderate to severe HS. CZP is approved for various inflammatory conditions but is unique because it does not cross the placenta actively. Data from pregnant women with rheumatic diseases exposed to CZP suggest that it does not pose an increased risk of fetal anomalies or fetal demise compared to the general population. Prospectively collected data from a pharmacovigilance safety database, which examined fetal and maternal outcomes in 1,137 women with rheumatic diseases exposed to CZP during pregnancy, indicate that there is no increased risk of fetal anomalies or fetal demise. These outcomes are consistent with those observed in the general population. This information is valuable for managing women with rheumatic diseases during pregnancy and offers an off-label treatment option for other inflammatory diseases like HS [10].

Etanercept

Adams et al. (2010) aimed to evaluate the efficacy and safety of etanercept, a dimeric human TNF receptor, in treating HS. In this double-blind, randomized study, patients were given a higher dose of etanercept for a longer period of time. The goal was to explain further the role etanercept played in HS therapy and to determine if the efficacy as well as the safety of the treatment are dose- or time-dependent. Etanercept, 50 mg, or placebo, was administered subcutaneously twice weekly for 12 weeks, followed by open-label etanercept, 50 mg, twice weekly for an additional 12 weeks. The study found no statistically significant difference between physician global assessment and Dermatology Life Quality Index (DLQI) at 12 or 24 weeks between treatment and placebo groups. The results suggest that future studies should focus on other agents for the treatment of HS. Etanercept is currently approved by the US FDA for treatment in the US of rheumatoid arthritis, juvenile idiopathic arthritis, psoriatic arthritis, psoriasis, and ankylosing spondylitis. Studies have also shown improvement in HS disease activity score and DLQI when using etanercept therapy. However, a recent study failed to find clinically significant efficacy of 50 mg/week subcutaneous administration of etanercept for 12 weeks [11].

Combination

Martin-Ezquerra et al. (2017) compared the effects of known anti-TNF drugs, including adalimumab, infliximab, and ustekinumab. It was determined that HS patients are likely to experience “flare-ups” of symptoms regardless of their treatment modality and that discontinuing any of these medications would result in a relatively quick relapse of disease symptoms. Only ~60% of HS patients using adalimumab have clinical responses without adverse effects, and only 50% of infliximab users experience reductions in lesion size and or severity. However, ustekinumab achieved symptom improvement rates as high as 82% in HS patients. These findings suggest that HS has multifactorial pathology, which likely requires more than anti-TNF therapy, such as antibiotics or retinoids, to achieve complete symptom reduction and prevent relapse from occurring. However, the role of TNF in HS pathology is still predominant based on the statistics mentioned above [12].

New Remedies

Schell et al. (2023) investigated the role of keratinocytes and epidermal immune cells in HS inflammation across all Hurley disease severity stages. The research found that HS lesional keratinocytes independently produce significant levels of chemokines, recruiting immune cells like neutrophils, CD8 T cells, and natural killer cells to the skin's epidermis. Keratinocytes were identified as the primary source of inflammatory cytokines in HS lesions, with limited contribution from dermal immune cells. When exposed to IFN-γ, keratinocytes expressed even more cytokines, including IL-1β, IL-12, IL-23, and IL-36γ. Importantly, the study showed that the Janus kinase (JAK) inhibitor ruxolitinib reduced the expression of inflammatory cytokines and chemokines in HS lesional keratinocytes. This suggests that ruxolitinib could be a potential topical treatment for HS, offering more effective results with fewer systemic side effects [13]. 

A retrospective study that determined the long-term (nine to 12 months) efficacy of a specific retinoid (acitretin, 0.59 mg/kg daily) monotherapy over 12 patients (four infertile women and eight men; mean age ~44.6 years) with severe treatment-resistant HS (in varying anatomical locations for over five years). Patients were previously treated with topical resorcinol (15%) cream in addition to systemic treatments such as antibiotics, methotrexate, and anti-TNF medications, and 11 of the 12 had also previously undergone surgery, including lesion removal and abscess aspiration. The same professional completed physical examinations and status surveys before and after treatment, and overall outcomes were determined via physician-global assessment focusing on reductions in inflammation. All 12 patients achieved remission, and a significant decrease in pain associated with nodules and or fibrosis, and four patients were reportedly pain-free (assessed using self-reporting via visual analog scale (VAS) before and after treatment). Additionally, long-lasting improvement was conveyed by nine of the 12 affected patients as they did not have symptom recurrence after six months (n = 1), one year (n = 3), >2 years (n = 2), >3 years (n = 2), and >4 years (n = 1), while the remaining three patients demonstrated moderate improvement. Clinical improvement began after two months of treatment, and further improvement became apparent after six months. After the treatment period, deep nodules and abscesses were absent, and there was a significant decrease in comedones, leaving only non-inflamed superficial nodules or pimples. Post-treatment, nine of the 12 patients had long-lasting improvement and or remission following discontinuation of acitretin (remissions ranged from six to 45 months), while the other three had a recurrence following discontinuation of specific retinoid therapy between one and three months [14].

Anti-IL-17 agents: Currently, the most encouraging biologic therapies in phase III trials for the treatment of HS include anti-IL-17 agents such as secukinumab and bimekizumab. Secukinumab is a fully human monoclonal antibody of the IgG1κ class that exhibits a high affinity for IL-17A. In a 24-week open-label pilot study involving nine patients with moderate to severe HS, secukinumab was administered subcutaneously at a dose of 300 mg weekly for five weeks, followed by doses every four weeks for the remaining 24 weeks. Although 78% of patients achieved an HiSCR by the 24th week, it was concluded that this dosing regimen used for psoriasis might not be optimal for HS due to higher inflammation levels. Another 24-week open-label trial included 20 patients with moderate to severe HS who received secukinumab at two different dose levels. After an initial dose of secukinumab 300 mg subcutaneously weekly for five weeks, nine patients received secukinumab 300 mg subcutaneously every four weeks, while 11 patients received secukinumab 300 mg subcutaneously every two weeks. A total of 70% of all patients achieved HiSCR by week 24. These results suggest that an increased frequency of secukinumab administration does not necessarily lead to improved clinical outcomes and may even raise the potential for side effects [15].

Additionally, there are three ongoing phase III randomized placebo-controlled trials aimed at evaluating the effectiveness and safety of two secukinumab dose regimens in HS patients. Bimekizumab, on the other hand, is a humanized monoclonal antibody of the IgG1κ class that can neutralize both IL-17A and IL-17F. A phase II clinical trial involved 90 patients with moderate to severe HS who were randomly assigned to receive bimekizumab, a placebo, or adalimumab. At week 12, 46% of patients treated with bimekizumab achieved a 75% reduction in total abscesses and nodule count (HiSCR 75), and 32% of them achieved an even higher HiSCR 90. In comparison, only 10% of patients in the placebo group achieved HiSCR 75, and none reached HiSCR 90. Among patients treated with adalimumab, 35% achieved HiSCR 75, and 15% achieved HiSCR 90. Presently, three phase III clinical studies are underway to assess the efficacy and safety of bimekizumab in the treatment of HS [15].

The initial clinical data on the effectiveness and safety of bimekizumab in inhibiting both IL-17A and IL-17F show promise as a viable treatment for HS. This dual inhibition approach has the potential to produce significant improvements in clinical outcomes and warrants further investigation. In a phase 2 randomized clinical trial involving 90 patients with HS (73 of whom completed the trial), bimekizumab consistently and significantly outperformed the placebo in terms of clinical outcomes in a double-blind, placebo-controlled setting. By week 12, bimekizumab exhibited a significantly higher rate of HiSCR compared to the placebo (57.3% versus 26.1%). Bayesian analysis showed a high posterior probability of superiority (0.998). Bimekizumab led to more substantial clinical improvements when compared to the placebo. Notably, at week 12, the mean International Hidradenitis Suppurativa Severity Score (IHS4) for bimekizumab-treated patients was 16.0 (SD 18.0), whereas it was substantially higher for those on placebo (mean (SD) IHS4, 40.2 (32.6)). Furthermore, bimekizumab-treated participants achieved significantly better results on rigorous outcome measures than the placebo. Specifically, at week 12, 46% of those receiving bimekizumab reached HiSCR75, and 32% reached HiSCR90, in contrast to the placebo group, in which only 10% achieved HiSCR75, and none reached HiSCR90. For those treated with adalimumab, 35% achieved HiSCR75, and 15% reached HiSCR90. These findings indicate that bimekizumab has the potential to be an effective treatment for HS, as it consistently demonstrated substantial improvements in clinical outcomes compared to the placebo, supporting the need for further research [15].

Anakinra is a recombinant IL-1 receptor antagonist that blocks the biological activity of naturally occurring IL-1 by completely inhibiting the binding of both IL-1⍺ and IL-1β to the IL-1 receptor. IL-1 is produced in response to various stimuli and is a major mediator of the inflammatory response. This study investigated whether anakinra would be a safe and efficacious approach to managing HS. The trial involved a 12-week treatment phase and follow-up phase for patients with HS and Hurley stage II or III HS. Twelve exclusion criteria were applied, including a history of systemic lupus erythematosus, rheumatoid arthritis, TNF-blocking therapy, vaccines, and hepatic dysfunction. Either a placebo or anakinra was administered subcutaneously. Patients were monitored at baselines, with six evaluations performed at each visit. The study assessed disease activity, sartorius score, and HiSCR at each visit. The HiSRC was defined as a 50% reduction in inflammatory lesion count and no increase in abscesses or draining fistulas compared to the baseline visit. The study found that the disease activity score decreased in 20% of placebo-treated patients compared to 78% of anakinra-treated patients at week 12. The HiSCR components were positive in 30% of patients randomized to placebo compared to 78% of those randomized to anakinra. There are no signs of setback after discontinuing anakinra, and results demonstrate that anakinra attenuates the severity of HS. The most effective treatment to this day for HS has been anti-TNF therapy, but not all patients respond to these interventions. Study results indicate that anakinra therapy has proven to be safe as an alternative HS treatment for patients, with injection site pain and mild infections being within the drug’s safety profile [16].

## Conclusions

The research on HS has provided significant insights into the complex pathophysiology of the condition and the effectiveness of various treatment options. A key focus has been the immunological dysregulation in HS, particularly the overproduction of proinflammatory cytokines like TNF-α, IL-17, IL-1, and IL-12/23, which has led to the development of targeted therapies. TNF inhibitors, such as adalimumab, have demonstrated notable success, with clinical response rates around 60-70%. However, high relapse rates following treatment discontinuation remain a challenge. CZP, an anti-TNP-α agent, shows promise for HS, especially in cases where patients are pregnant. The efficacy of etanercept, another TNF inhibitor, is less clear due to inconsistent results across studies.

Emerging treatments for HS are showing promise. Anti-IL-17 agents like secukinumab and bimekizumab have reported response rates of up to 82% in some studies. The JAK inhibitor ruxolitinib shows potential as a topical treatment, reducing inflammatory cytokine production in keratinocytes. Trials with the IL-1 receptor antagonist anakinra have also yielded positive results, offering a safe and effective alternative for HS patients. Acitretin, a retinoid, has shown significant long-term efficacy in treating severe, treatment-resistant HS, with many patients experiencing prolonged periods of remission. Additionally, combination therapies that integrate biologics with antibiotics, retinoids, or surgical approaches are likely to become more common as the understanding of HS's multifactorial pathogenesis grows. Combining therapies that target different pathways may also provide better symptom control and reduce relapse. Further large-scale clinical trials and long-term studies will be crucial to confirming the effectiveness and safety of these emerging treatments.
